# Proximal Junction Kyphosis in Adult Scoliosis: Best Postoperative Radiological Predictors—A Retrospective Cohort Study

**DOI:** 10.1155/2022/9814416

**Published:** 2022-03-23

**Authors:** Daudi R. Manini, Hong-qi Zhang, Qile Gao, Shao-hua Liu, Wang YuXiang, YuXuan Du

**Affiliations:** ^1^Department of Spine Surgery, Xiangya Hospital of Central South University, Changsha 410008, Hunan, China; ^2^Department of Orthopedic Surgery, Mwananyamala Regional Referral Hospital, P.O. Box 61665, Dar-es-Salaam, Tanzania

## Abstract

**Background:**

Proximal junction kyphosis (PJK) is the postsurgical radiographic event seen in the surgical plane after the fusion of a spinal deformity. Unfavorable health outcomes have been reported in symptomatic PJK patients compared to non-PJK patients.

**Methods:**

The data for adult scoliosis patients who underwent curve correction were extracted from the hospital database. Pelvic and spinal parameters were measured and calculated to compare four predictive formulae for occurrences of PJK. Formula 1. Restoration of hypothetical values of lumbar lordosis (LL) and thoracic kyphosis (TK) according to pelvic incidence (PI). Formula 2. Evaluation of global sagittal alignment. Formula 3. Restoration of the apex of LL to its hypothetical position according to the spine shape. Formula 4. Evaluation of positive-sum and negative-sum of (LL + TK).

**Results:**

A total number of cases were 52. There were 14 cases of PJK. The incidence of PJK was 26.9%, and the mean age for PJK cases was 63.2 ± 5.2. The excellent predictor for occurrences of PJK was formula 3. Postsurgical sagittal apexes of lumbar lordosis were located in their hypothetical position in 24 cases, and 12.5% of these cases developed PJK. While sagittal apexes were not located in their hypothetical position in 28 patients, PJK occurred in 39.3% of them (*P*=0.03, OR: 4.53, (95% CI: 1.09–18.9)). The second good predictor for occurrences of PJK was formula 2 (GSA >45° versus GSA <45° OR = 2.5, (95% CI: 0.67–9.38), *P*=0.17). The other two formulae (1 and 4) were not good predictors for occurrences of PJK.

**Conclusion:**

Among the four proposed formulae for predicting occurrences of PJK, the position of the sagittal apex of lumbar lordosis is an excellent predictor of the development of PJK, followed by GSA. Hypothetical values of LL and TK, and positive or negative-sum of (LL + TK), are weak predictors for occurrences of PJK.

## 1. Introduction

Proximal junction kyphosis (PJK) is the postsurgical radiographic event seen in the surgical plane after the fusion of a spinal deformity. In wide view, PJK is regarded as a form of adjacent segment disease associated with spinal fusion, mostly occurring after scoliosis or kyphosis surgery [1, 2]. Sometimes PJK may associate with clinical symptoms and sometimes is merely radiographic findings [3]. PJK frequently arises just above the uppermost instrumented vertebrae (UIV). The most accepted PJK definition is the one defined by Glattes et al. [4]. The PJK angle is the angle subtended by the lower endplate of the UIV to the upper endplate of two vertebrae proximal. PJK is said to be present if the sagittal Cobb angle is ≥10° and at least 10° larger than the preoperative measurement.

The authors have proposed several formulae that help to predict the occurrences of PJK immediately after curve correction. Among the proposed formulae are the following:

1. Restoration of the theoretical value of lumbar lordosis (LL) and thoracic kyphosis (TK) according to pelvic incidence (PI) [5]
2. Restoration of global sagittal alignment (GSA), GSA = TK + LL + PI [2]
3. Restoration of the sagittal apex of the LL at the L4 vertebra for type 1 and type 2 lordosis curve, and at the L3 vertebra or L3–L4 disc for type 3 and type 4 lordosis curve [6]
4. When the magnitude of TK is larger than the magnitude of LL (TK > LL) [7]

Among the above 4 proposed formulae, it is not known which formula is superior to others in predicting the occurrences of PJK. Furthermore, there is no common agreement on direct postoperative radiographic prognostic criteria for PJK that justify close monitoring of the patients. We hypothesize that the future occurrences of PJK after curve correction can be determined immediately postoperatively by assessing the radiographic variables (TK, LL, PI, and GSA) using the correct formula.

The aim of this retrospective study is to assess which immediate postsurgical radiographic variables using the above proposed formulae are the best in forecasting the future occurrences of PJK in adult scoliosis after deformity correction.

## 2. Materials and Methods

A retrospective study of 52 adult scoliosis patients who underwent scoliosis curve correction in Xiangya Hospital of Central South University from January 2012 to December 2020 was carried out. After approval from the institutional review board of Xiangya Hospital of Central South University, the data were retrieved from the hospital database.

### 2.1. Inclusion Criteria

The inclusion criteria were as follows: (1) Patient with scoliosis deformity Cobb ≥25° on the anteroposterior (AP) view. (2) Patients aged ≥25 years. (3) Patients with a follow-up period of at least 2 years. (4) Patients with all radiological films (preoperative, immediate postoperative, and last follow-up films) available. (5) Patients with lower instrumented vertebra end at L4 or below.

### 2.2. Exclusion Criteria

The exclusion criteria were as follows: (1) Patients with surgery of other spinal abnormalities such as tumor, tuberculosis, or accident. (2) Patients with incomplete radiographic data. (3) Patients with a follow-up of fewer than 6 months.

### 2.3. Surgical Procedure

All cases underwent a one-stage posterior-only approach performed by senior spinal surgeons of our department. The surgical technique included the anatomical exposure of the posterior bony structure with a subperiosteal dissection to reduce blood loss and curve stiffness. Then, the insertion of bilateral pedicle screws or hooks based on preoperative surgical planning. In general, pedicle screws were inserted at the levels below T6, and hooks were used in the upper segments. The size and flexibility of the curve observed during surgery helped the surgeon to decide the need for an osteotomy or not. In the rigid segments, the costotransverse joint was released by resecting the bilateral transverse processes. If necessary, parts of the ribs and transverse processes at the apex vertebra were removed. And in the lumbar regions, pedicle subtraction osteotomy (PSO) was used in some cases. Precontoured rods were inserted in the convex and concave sides of the curve. The correction technique involved the combination of derotation, translation, compression, and distraction maneuvers. Two or more cross-linkers were used to enhance stability. Local autologous bone grafts harvested from the posterior elements were used for arthrodesis. Finally, the drainage catheters were placed and the incision was closed in layers.

Somatosensory evoked potential (SSEP) and wake-up tests were used to monitor neurological functions intraoperatively.

#### 2.3.1. Formula 1

Hypothetical values of LL and TK were calculated as previously stated by Vialle et al. [5] LL = 0.67PI + 23.7 and TK = 0.15PI + 43. Then, the rate of occurrences of PJK in patients whose immediate postoperative LL and TK values were equal or nearly equal to their theoretical value were compared to patients whose LL and TK values deviated far from their theoretical value (a discrepancy of 3° of a real value from its theoretical value was accepted).

#### 2.3.2. Formula 2

Immediate postoperative GSA was calculated by the formula GSA = LL + TK + PI, as previously reported by Yagi et al. [2]. And the occurrence of PJK in patients with GSA >45° versus GSA <45° was compared.

#### 2.3.3. Formula 3

The position of the sagittal apex of LL was marked on immediate postoperative radiological films. The theoretical apex of lumbar lordosis was considered to be L4 for patients with PI < 55° and L3 for patients with PI > 55° as stated by Roussouly et al. [6]. Then, the occurrences of PJK among patients whose sagittal apexes of LL were at their theoretical position were compared to patients whose sagittal apexes of LL were not at their theoretical position.

#### 2.3.4. Formula 4

The sum of LL and TK was calculated from the immediate postoperative radiological films as stated by Mendoza et al. [7]. The occurrences of PJK between positive-sum (LL + TK) and negative-sum (LL + TK) were compared.

The PJK angle was calculated from radiological films in the final follow-up visit as previously described by Glattes et al. [4].

### 2.4. Statistical Analysis

IBM SPSS (version 21) was used for statistical analysis. Fisher's exact test was performed for continuous variables, and the Chi-square test was used for noncontinuous variables. The odds ratio (OR) was calculated. A *P* value of <0.05 was considered to be statistically significant.

## 3. Results

Fifty-two patients fulfilled our inclusion criteria; the female-to-male ratio was 16.3 : 1. The mean age for all cases was 61.1 ± 6.3. The mean follow-up time in years was 2.05 ± 0.85. The mean number of vertebral levels fused for PJK and non-PJK groups was 10.14 ± 2.03 and 9.79 ± 2.51 (*P*=0.66), respectively. The mean time in years for the occurrences of PJK was 1.25 ± 0.61 (0.5–3), with 71.43% of cases developing PJK within 1 year postoperatively. There were 14 cases of PJK. The incidence of PJK was 26.9%, and the mean age for PJK cases was 63.2 ± 5.2. The UIV was located in the upper thoracic (T1–T4) in 67.3% of cases, in the midthoracic (T5–T9) in 25% of cases, and in the thoracolumbar (T10–L2) in 7.7% of cases. The rate of occurrences of PJK in these regions were 25.71%, 30%, and 25% (*P*=0.94), respectively. The preoperative LL apex was located at L2 in 1.92% of patients, the L2–L3 disc in 21.15% of patients, the L3 in 26.92% of patients, the L3–L4 disc in 25% of patients, and the L4 in 25% of patients. Other results for spinal pelvic parameters are shown in Table 1.

The occurrences of PJK as speculated by the above-mentioned 4s formulae are as follows:

### 3.1. Formula 1

The mean theoretical value of LL as calculated by Vialle's method was −60.31 ± 9.31, different from the observed postoperative LL value of −54.25 ± 11.41 (*P*=0.004). And the mean theoretical value of TK was 51.2 ± 2.08, significantly different from the observed postoperative value of 44.54 ± 14.91 (*P*=0.004). PJK occurred in 26% of patients with postoperative LL and Tk values equal to their theoretical values, and in 27.6% of patients with LL and TK different from the observed postoperative values (odds ratio (OR): 1.08, 95% confidence interval (CI): (0.31–3.72), *P*=0.9).

### 3.2. Formula 2

The mean GSA was 44.93 ± 18.6. There were 22 patients (44.23%) with GSA <45° and 30 patients (55.77%) with GSA >45%. PJK occurred in 16% of patients with GSA <45° and 34.5% of patients with GSA >45° (OR = 2.5, (95% CI, 0.67–9.38), *P*=0.17).

### 3.3. Formula 3

Postsurgical sagittal apexes of lumbar lordosis were located in their theoretical position in 24 patients, and 12.5% of these patients developed PJK. While sagittal apexes were not located in their theoretical position in 28 patients, 39.3% of these patients developed PJK (*P*=0.03, OR: 4.53, (95% CI: 1.09–18.9)).

### 3.4. Formula 4

The mean value of LL + TK was −9.7 ± 17.4. The LL + TK value was positive in 14 patients, and 28.6% of these patients developed PJK. While the LL + TK value was negative in 38 patients, 26.3% of the patients developed PJK (OR = 1.12, 95%CI: (0.29–4.31), *P*=0.87).

**Figure 1:**
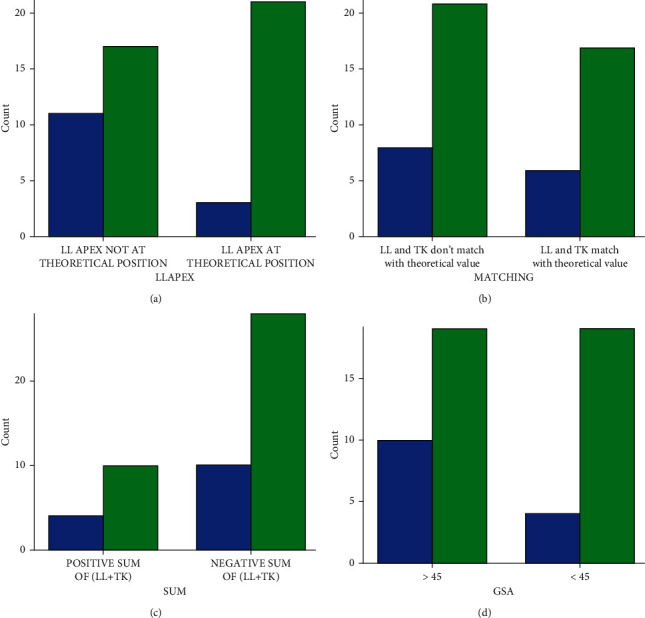
Compound bar charts (a–d) show the occurrences of PJK as predicted by each formula. LL = lumbar lordosis, TK = thoracic kyphosis, GSA = global sagittal alignment, and PJK = proximal junctional kyphosis. The blue bar shows the number of patients who developed PJK, while the green bar shows the number of patients who did not develop PJK.

The results for each formula as it predicts the occurrences of PJK are shown in Figure 1.

## 4. Discussion

Proximal junction kyphosis (PJK) is the postsurgical radiographic event seen in the surgical plane after the fusion of a spinal deformity. PJK can be diagnosed in the radiographic film as soon as 8 weeks postoperatively [8, 9]. The incidence of PJK as reported in the literature varies greatly and ranges from 5% to 46% in spinal patients who undergo deformity curve correction [2, 10]. The incidence of PJK in our study was 26.9%, similar to the incidence reported by other researchers [10, 11].

Several risk factors for occurrences of PJK have been reported in the literature including age more than 55 years, large abnormal preoperative sagittal parameters (high lumbar lordosis and long thoracic kyphosis), application of pedicle screws, thoracoplasty, posterior and combined anteroposterior spinal fusion surgeries, fusion involving the lower lumbar spine and sacrum, osteoporosis, and a high body mass index [12, 13]. To date, there is no common agreement on direct postoperative radiographic prognostic criteria for PJK that has been established to monitor the patients at risk in clinical practice. Some researchers have proposed that the occurrences of PJK could be evaluated by the use of either preoperative or postoperative radiographic parameters. A retrospective study by Lee et al.'s [14] to determine the possible cause of PJK in adolescent idiopathic scoliosis patients, the authors proposed that the occurrences of PJK could be predicted from preoperative radiographic films. Also, Maruo et al. [15] suggested that improved postoperative radiographic sagittal alignment parameters are an important method to mitigate the development of PJK after adult deformity correction.

In this retrospective study, we have used four formulae, to predict the occurrences of PJK based on immediate postoperative radiological films. We found that among the four proposed formulae, the position of the sagittal apex of lumbar lordosis (formula 3) is the best immediate postoperative radiographic predictor of occurrences of PJK among the four hypothesized formulae. It has a great predictive value (OR: 4.5; (95% CI: 1.09–18.9)). The OR 4.5 means that the occurrence of PJK is almost 5 times high when the apex of LL is not at its theoretical position compared to when it is at its theoretical position. A similar finding has been reported in the literature by other scholars [10]. Roussouly et al. [6] after studying the sagittal alignment of 160 healthy volunteers, divided the spine into four types based on inflexion point. Type 1 and type 2 had low PI and type 3 and type 4 had high PI. They found that the lower arc of lordosis is a key determinant of global lordosis (lordosis tilt angle, location of the apex, and the number of lordotic vertebra). Both low SS and low PI are accompanied by a flat and short lumbar lordosis, while high SS and high PI are accompanied by a long and curved lumbar lordosis. They further proposed that the spine is well balanced when the lordosis apex is at L4 for low PI and L3 for high PI. In our study, the occurrences of PJK were very high when the apexes of sagittal lordosis were not at their theoretical position compared to when they were positioned at their theoretical position. This may be explained that when the postoperative apex is located high above its theoretical position kyphosis has less room to be constructed and increase the risk of occurrences of PJK and other spinal abnormalities [16].

The second good predictor for the occurrences of PJK in our study was GSA (formula 2). The predictive value for GSA was OR = 2.5, (95% CI, 0.67–9.38). This means that when GSA is >45°, the occurrence of PJK is 2.5 times as compared to when GSA is <45°. Lafage et al. [17] in their virtual model stated that realignment surgery for an adult spinal deformity (ASD) can be achieved by restoring a balanced sagittal alignment. Yagi et al. [2] in their retrospective review of 157 cases, they found that more than 80% of patients with non-ideal global sagittal alignment (GSA >45°) developed PJK. Sebaaly et al. [10] in their retrospective study of 250 cases of adult idiopathic scoliosis from a multicentric database, found that GSA >45° was associated with the occurrences of PJK, with OR 1.7. In this study, we also found that GSA >45° is highly associated with occurrences of PJK, with OR 2.5. This might be explained by the reason that increased GSA results in the positive (SVA) which increases the stress on the proximal and distal junctions of the instrumentation and leads to the occurrences of PJK [18].

The positive and negative sum of LL and TK in formula 4 was not sensitive in predicting the occurrences of PJK (OR: 1.12, (95% CI: 0.29–4.39)). We have traced back to the derivation of this formula and found that formula 4 was derived from a small sample size of only 54 patients, and even the results for the multivariate model that led to the generation of formula 4 had an odds ratio of 0.861 which is still weak in decision making [7].

The hypothetical values of LL and TK for formula 1 were also weak in predicting the occurrences of PJK. This is probably because this formula was derived from healthy volunteers with a small sample size of 300 participants from a single center. Moreover, the R^2^s obtained from the regression model were 0.74 and 0.44, respectively, for LL and TK, which is still a weak correlation [5].

Despite some of the patients' parameters meeting all the criteria of the abovementioned formulae, PJK occurred, and even formulae 1, 2, and 4 failed to reject the null hypothesis as the CI included 1. This might be explained by the reason that the risk factors for occurrences of PJK are many. And even some factors have been reported in the literature, including age, low bone mineral density, the use of pedicle screws, thoracoplasty, combined anteroposterior surgery, and long fusion surgery [19–21].

**Figure 2:**
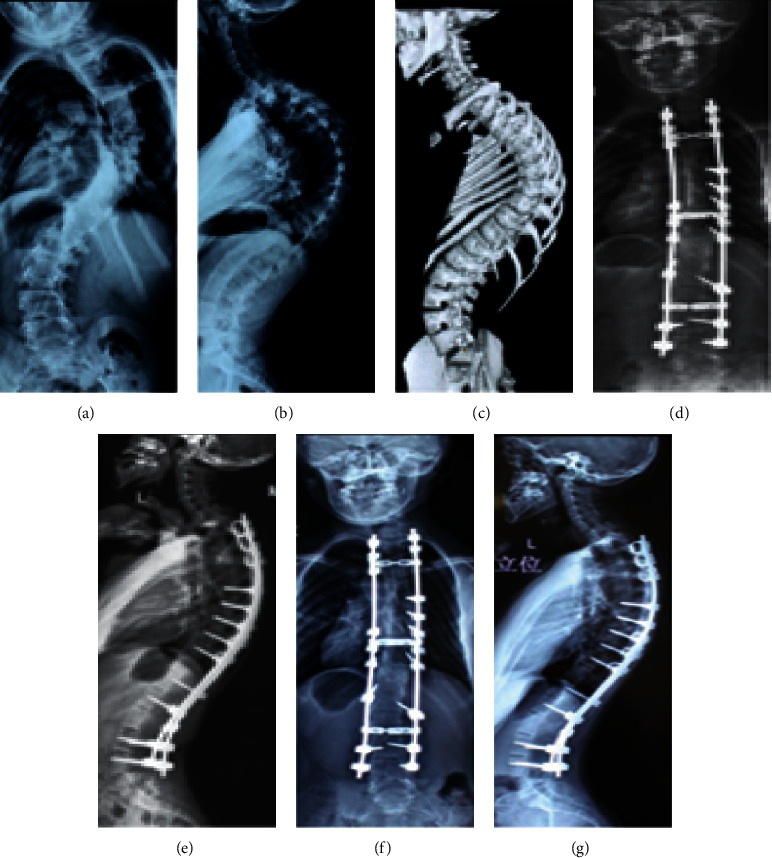
Radiographic films of a 40-year-old female patient with neglected scoliosis who underwent scoliosis curve correction and developed asymptomatic PJK after one year. (a) Preoperative anteroposterior (AP) X-ray views; (b) preoperative lateral X-ray view; and (c) a preoperative bone scan 3D model. (d, e) Immediate postoperative AP and lateral X-ray films and (f, g) AP and lateral X-ray films conducted after 1 year. The PJK angle after 1 year was 110 greater than immediate postoperative.

In this study, PJK was only a radiological finding; none of our patients developed PJK symptoms such as pain, neurological deficit, low SRS score, or proximal junction failure (Figure 2). So we still agree with other authors [2, 22, 23] who reported no significant clinical symptoms between PJK patients and non-PJK patients.

The most important finding of this study is that the theoretical position of the sagittal apex of lumbar lordosis is a preventive factor for the development of PJK. This finding can guide the surgeon to decide the level of pedicle subtraction osteotomy (PSO) or a suitable place to put lordotic cages during spine deformity correction.

This study has some weaknesses which are as follows: a retrospective study design with some data missing and some patients who lost follow-up. For example, the data for bone mineral density and Oswestry disability index scores were not found. It also is a single-center study with a small sample size. But despite these limitations, the results from this study are similar to those reported by other scholars [10]. We also call upon further studies to investigate the reliability and repeatability of both formulae 1 and 4 in predicting the occurrences of PJK.

## 5. Conclusion

Among the four proposed formulae for predicting occurrences of PJK, the position of the sagittal apex of lumbar lordosis is an excellent predictor of the development of PJK, followed by GSA. Hypothetical values of LL and TK, and the positive or negative-sum of (LL + TK), are weak predictors for occurrences of PJK.

## Figures and Tables

**Table 1:** Preoperative and postoperative spinal pelvic parameters in degree.

	Preoperative		Postoperative		*P* value
	Mean	SD	Mean	SD	
Pelvic incidence	54.64	13.91			
Pelvic tilt	22.98	8.08	19.28	7.64	0.018
Lumbar lordosis	−41.26	10.95	−54.25	11.41	≤0.001
Thoracic kyphosis	39.76	12.17	44.54	14.91	0.076
Sacral slope	29.99	10.44	33.84	9.73	0.054

SD = standard deviation.

## Data Availability

All the datasets analyzed during this study are included in this article, given as additional file 1.

## Supplementary Materials

Supplementary Materials Additional file 1. Microsoft Excel worksheet (.xlsx) containing data used in analysis for this study.
